# On the Relationship between Variational Level Set-Based and SOM-Based Active Contours

**DOI:** 10.1155/2015/109029

**Published:** 2015-04-19

**Authors:** Mohammed M. Abdelsamea, Giorgio Gnecco, Mohamed Medhat Gaber, Eyad Elyan

**Affiliations:** ^1^Department of Mathematics, Faculty of Science, University of Assiut, Assiut 71516, Egypt; ^2^IMT Institute for Advanced Studies, Piazza S. Francesco 19, 55100 Lucca, Italy; ^3^Robert Gordon University, Garthdee House, Garthdee Road, Aberdeen AB10 7QB, UK

## Abstract

Most Active Contour Models (ACMs) deal with the image segmentation problem as a functional optimization problem, as they work on dividing an image into several regions by optimizing a suitable functional. Among ACMs, variational level set methods have been used to build an active contour with the aim of modeling arbitrarily complex shapes. Moreover, they can handle also topological changes of the contours. Self-Organizing Maps (SOMs) have attracted the attention of many computer vision scientists, particularly in modeling an active contour based on the idea of utilizing the prototypes (weights) of a SOM to control the evolution of the contour. SOM-based models have been proposed in general with the aim of exploiting the specific ability of SOMs to learn the edge-map information via their topology preservation property and overcoming some drawbacks of other ACMs, such as trapping into local minima of the image energy functional to be minimized in such models. In this survey, we illustrate the main concepts of variational level set-based ACMs, SOM-based ACMs, and their relationship and review in a comprehensive fashion the development of their state-of-the-art models from a machine learning perspective, with a focus on their strengths and weaknesses.

## 1. Introduction

Image segmentation is the problem of partitioning the domain *Ω* of an image *I*(*x*), where *x* ∈ *Ω* is the pixel location within the image, into different subsets *Ω*_*i*_, where each subset has a different characterization in terms of color, intensity, texture, and/or other features used as similarity criteria. Segmentation is a fundamental component of image processing and plays a significant role in computer vision, object recognition, and object tracking.

Traditionally, image segmentation methods can be classified into five categories. The first category is made up of threshold-based segmentation methods [1]. These methods are pixel-based and usually divide the image into two subsets, that is, the foreground and the background, using a threshold on the value of some feature (e.g., gray level and color value). These methods assume that the foreground and background in the image have different ranges for the values of the features to be thresholded. Over the years, many different thresholding techniques have been developed, including Minimum error thresholding, Moment-preserving thresholding, and Otsu's thresholding, just to mention a few. The most popular thresholding method, Otsu's algorithm [2], improves the image segmentation performance over other threshold-based segmentation methods in the following way. The threshold used in Otsu's algorithm is chosen in such a way to optimize a trade-off between the maximization of the interclass variance (i.e., between pairs of pixels belonging to the foreground and the background, resp.) and the minimization of the intraclass variance (i.e., between pairs of pixels belonging to the same region). Otsu's thresholding algorithm and its extension to the case of multiple thresholds [3] are good for thresholding an image whose intensity histogram is either bimodal or multimodal (e.g., they provide a satisfactory solution in the case of the segmentation of large objects with nearly uniform intensities, significantly different from the intensity of the background). However, they have not the ability to segment images with a unimodal distribution (e.g., images containing small objects with different intensities), and their outputs are sensitive to noise. Thus, postprocessing operations are usually required to obtain a final satisfactory segmentation.

The second category of methods is called boundary-based segmentation [4]. These methods detect boundaries and discontinuities in the image based on the assumption that the intensity values of the pixels linking the foreground and the background are distinct. The first/second order derivatives of the image intensity are usually used to highlight those pixels (e.g., this is the case of Sobel and Prewitt edge detectors [4] as first-order methods, and the Laplace edge detector [1] as a second-order method, resp.). The difference between first and second order methods is that the latter can also localize the local displacement and orientation of the boundary. By far the most accurate technique of detecting boundaries and discontinuities in an image is the Canny edge detector [5]. The Canny edge detector is less sensitive to noise than other edge detectors, as it convolves the input image with a Gaussian filter. The result is a slightly blurred version of the input image. This method is also very easy to be implemented. However, it is still sensitive to noise and leads often to a segmentation result characterized by a discontinuous detection of the object boundaries.

The third category of methods is called region-based segmentation [6]. Region-based segmentation techniques divide an image into subsets based on the assumption that all neighboring pixels within one subset have a similar value of some feature, for example, the image intensity. Region growing [7] is the most popular region-based segmentation technique. In region growing, one has to identify at first a set of seeds as initial representatives of the subsets. Then, the features of each pixel are compared to the features of its neighbor(s). If a suitable predefined criterion is satisfied, then the pixel is classified as belonging to the same subset associated with its “most similar” seed. Accordingly, region growing relies on the prior information given by the seeds and the predefined classification criterion. A second popular region-based segmentation method is region “splitting and merging.” In such method, the input image is first divided into several small regions. Then, on the regions, a series of splitting and merging operations are performed and controlled by a suitable predefined criterion. As region-based segmentation is an intensity-based method, the segmentation result in general leads to a nonsmooth and badly shaped boundary for the segmented object.

The fourth category of methods is learning-based segmentation [8]. There are two general strategies for developing learning-based segmentation algorithms, namely, generative learning and discriminative learning. Generative learning [9] utilizes a data set of examples to build a probabilistic model, by finding the best estimate of its parameters for some prespecified parametric form of a probability distribution. One problem with these methods is that the best estimate of the parameters may not provide a satisfactory model, because the parametric model itself may not be correct. Another problem is that the classification/clustering framework associated with a parametric probabilistic model may not provide an accurate description of the data due to the limited number of parameters in the model, even in the case in which its training is well performed. Techniques following the generative approach include K-means [10], the Expectation-Maximization algorithm [11], and Gaussian Mixture Models [12]. Discriminative learning [13, 14] ignores probability and attempts to construct a good decision boundary directly. Such an approach is often extremely successful, especially when no reasonable parametric probabilistic model of the data exists. Discriminative learning assumes that the decision boundary comes from another class of nonparametric solutions and chooses the best element of that class according to a suitable optimality criterion. Techniques following the discriminative approach include Linear Discriminative Analysis [15], Neural Networks [16], and Support Vector Machines [17]. The main problems with the application of these methods to image segmentation are their sensitivity to noise and the discontinuity of the resulting object boundaries.

The last category of methods is energy-based segmentation [18, 19]. This class of methods is based on an energy functional and deals with the segmentation problem as a functional optimization problem, whose goal is to partition the image into regions based on the maximization/minimization of the energy functional. (Loosely speaking, a functional is defined as a function of a function, that is, a functional takes a function as its input argument and returns a scalar.) The most well-known energy-based segmentation techniques are called “active contours” or Active Contour Models (ACMs). The main idea of active contours is to choose an initial contour inside the image domain to be segmented and then make such a contour evolve by using a series of shrinking and expanding operations. Some advantages of the active contours over the aforementioned methods are that topological changes of the objects to be segmented can be often handled implicitly. More importantly, complex shapes can be modeled without the need of prior knowledge about the image. Finally, rich information can be inserted into the energy functional itself (e.g., boundary-based and region-based information).

More specifically, ACMs usually deal with the segmentation problem as an optimization problem, formulated in terms of a suitable “energy” functional, constructed in such a way that its minimum is achieved in correspondence with a contour that is a close approximation of the actual object boundary. Starting from an initial contour, the optimization is performed iteratively, evolving the current contour with the aim of approximating better and better the actual object boundary (hence the denomination “active contour” models, which are used also for models that evolve the contour but are not based on the explicit minimization of a functional [20]). In order to guide efficiently the evolution of the current contour, ACMs allow to integrate various kinds of information inside the energy functional, such as local information (e.g., features based on spatial dependencies among pixels), global information (e.g., features which are not influenced by such spatial dependencies), shape information, prior information, and a-posteriori information learned from examples. (Due to the possible lack of a precise prior information on the shape of the objects to be segmented, in this respect most ACMs make only the assumption that it is preferable to have a smooth boundary [21]. This goal is achieved by incorporating a suitable regularization term into their energy functionals [19].) As a consequence, depending on the kind of information used, one can divide ACMs into several categories, for example, edge-based ACMs [22–25], global region-based ACMs [26, 27], edge/region-based ACMs [28–30], local region-based ACMs [31–33], and global/local region-based ACMs [34, 35]. In particular, edge-based ACMs make use of an edge-detector (in general, the gradient of the image intensity) to stop the evolution of the active contour on the true boundaries of the objects of interest. Instead, region-based ACMs use with the same purpose statistical information about the regions to be segmented (e.g., intensity, texture, and color distribution). Depending on how the active contour is represented, one can also distinguish between parametrized [36] and variational level set-based ACMs [26]. One important advantage of the latter is that they can handle implicitly topological changes of the objects to be segmented.

Although ACMs often provide an effective and efficient means to extract smooth and well-defined contours, trapping into local minima of the energy functional may still occur, because such a functional may be constructed on the basis of simplified assumptions on properties of the images to be segmented (e.g., the assumption of Gaussian intensity distributions for the sets *Ω*_*i*_ in the case of the Chan-Vese active contour model [21, 26]). Motivated by this observation and by the specific ability of SOMs to learn, via their topology preservation property [37], information about the edge map of the image (i.e., the set of points obtained by an edge-detection algorithm), a new class of ACMs, named SOM-based ACMs [38, 39], has been proposed with the aim of modelling and controlling effectively the evolution of the active contour by a Self-Organizing Map (SOM), often without relying on an explicit energy functional to be minimized. In this paper, we review some concepts of ACMs with a focus on SOM-based ACMs, illustrating both their strengths and limitations. In particular, we focus on variational level set-based ACMs and SOM-based ACMs, and on their relationship. The paper is a substantial extension of the short survey about SOM-based ACMs that we presented in [40]. A summary of the main strengths and drawbacks of the ACMs presented in the survey is reported in Table 1. Illustrating the motivations for such strengths and drawbacks is the main focus of this paper.

The paper is organized as follows.  Section 2 provides a summary of variational level set-based ACMs. In Section 3, we review the state of the art of SOM-based ACMs not used in combination with variational level set methods.  Section 4 describes a recent class of SOM-based ACMs combined with such methods. Finally, Section 5 provides some conclusions.

## 2. Variational Level Set-Based ACMs

To build an active contour, there are mainly two methods. The first one is an explicit or Lagrangian method, which results in parametric active contours, also called “Snakes” from the name of one of the models that use such a kind of parametrization [19]. The second one is an implicit or Eulerian method, which results in geometric active contours, known also as variational level set methods.

**Figure 1:**
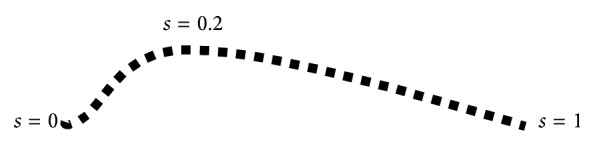
The parametric representation of a contour.

In parametric ACMs, the contour *C*, see Figure 1, is represented as

(1) $$\begin{array}{c} C∶=\left\{x\in \Omega :x=\left(x_{1}\left(s\right),x_{2}\left(s\right)\right),0\leq s\leq 1\right\}, \end{array}$$

where *x*_1_(*s*) and *x*_2_(*s*) are functions of the scalar parameter *s*. A representative parametric ACM is the Snakes model, proposed by Kass et al. [19] (see also [36] for successive developments).

The main drawbacks of parametric ACMs are the frequent occurrence of local minima in the image energy functional to be optimized (which is mainly due to the presence of a gradient energy term inside such a functional), and the fact that topological changes of the objects (e.g., merging and splitting) cannot be handled during the evolution of the contour.

**Figure 2:**
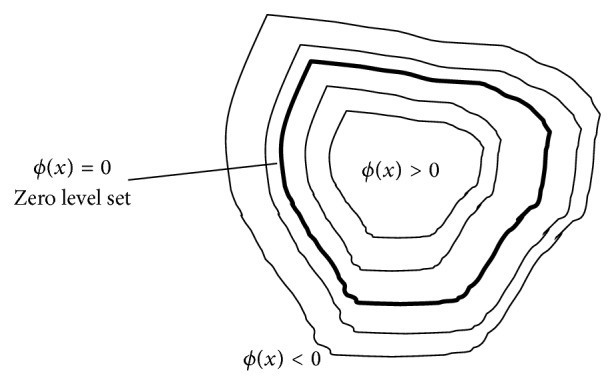
The geometric representation of a contour.

The difference between parametric active contour and geometric (or variational level set-based) Active Contour Models is that in geometric active contours, the contour is implemented via a variational level set method. Such a representation was first proposed by Osher and Sethian [41]. In such methods, the contour *C*, see Figure 2, is implicitly represented by a function *ϕ*(*x*), called “level set function,” where *x* is the pixel location inside the image domain *Ω*. The contour *C* is then defined as the zero level set of the function *ϕ*(*x*), that is,

(2) $$\begin{array}{c} C∶=\left\{x\in \Omega :\phi \left(x\right)=0\right\}. \end{array}$$

A common and simple expression for *ϕ*(*x*), which is used by most authors, is

(3) $$\begin{array}{c} \phi \left(x\right)∶=\left\{\begin{array}{cc} +\rho , & \text{for}\;x\in \text{inside}\left(C\right),\\ 0, & \text{for}\;x\in C,\\ -\rho , & \text{for}\;x\in \text{outside}\left(C\right), \end{array}\right. \end{array}$$

where *ρ* is a positive real number (possibly dependent on *x* and *C*, in such case it is denoted by *ρ*(*x*, *C*)).

In the variational level set method, expressing the contour *C* in terms of the level set function *ϕ*, the energy functional to be minimized can be expressed as follows:

(4) $$\begin{array}{c} E\left(\phi \right)∶=E_{\text{in}}\left(\phi \right)+E_{\text{out}}\left(\phi \right)+E_{C}\left(\phi \right), \end{array}$$

where *E*_in_(*ϕ*) and *E*_out_(*ϕ*) are integral energy terms inside and outside the contour, and *E*_*C*_(*ϕ*) is an integral energy term for the contour itself. More precisely, the three terms are defined as

(5)  Ein(ϕ)∶=∫ϕx>0e(x)dx=∫ΩHϕx·exdx, Eout(ϕ)∶=∫ϕx<0e(x)dx=∫Ω1−Hϕx·exdx, EC(ϕ)∶=∫Ω∇H(ϕ(x))dx=∫Ωδ(ϕ(x))·∇ϕ(x)dx,

where *e*(*x*) is a suitable loss function, and *H* and *δ* are, respectively, the Heaviside function and the Dirac delta distribution, that is,

(6) $$\begin{array}{c} H\left(z\right)∶=\left\{\begin{array}{cc} 1, & \text{if}\;z\geq 0,\\ 0, & \text{if}\;z<0, \end{array}\right.\\ \delta \left(z\right)∶=\frac{d}{dz}H\left(z\right). \end{array}$$

Accordingly, the evolution of the level set function *ϕ* provides the evolution of the contour *C*. In the variational level set framework, the (local) minimization of the energy functional *E*(*ϕ*) can be obtained by evolving the level set function *ϕ* according to the following Euler-Lagrange Partial Differential Equation (in the following, when writing partial differential equations, in general we do not write explicitly the arguments of the involved functions, which are described either in the text, or in the references from which such equations are reported) (PDE):

(7) $$\begin{array}{c} \frac{\partial \phi }{\partial t}=-\frac{\partial E\left(\phi \right)}{\partial \phi }, \end{array}$$

where *ϕ* is now considered as a function of both the pixel location *x* and time *t*, and the term ∂*E*(*ϕ*)/∂*ϕ* denotes the functional derivative of *E* with respect to *ϕ* (i.e., loosely speaking, the generalization of the gradient to an infinite-dimensional setting). So, (7) represents the application to the present functional optimization problem of an extension to infinite dimension of the classical gradient method for unconstrained optimization. According to the specific kind of PDE (see (7)) that models the contour evolution, variational level set methods can be divided into several categories, such as* Global Active Contour Models* (GACMs) [42–46], which use global information, and* Local Active Contour Models* (LACMs) [47–51], which use local information.

### 2.1. Unsupervised Models

In order to guide efficiently the evolution of the current contour, ACMs allow to integrate various kinds of information inside the energy functional, such as local information (e.g., features based on spatial dependencies among pixels), global information (e.g., features that are not influenced by such spatial dependencies), shape information, prior information, and also a-posteriori information learned from examples. As a consequence, depending on the kind of information used, one can further divide ACMs into several subcategories, for example, edge-based ACMs [22–25, 52, 53], global region-based ACMs [26, 27, 45, 54, 55], edge/region-based ACMs [28, 30, 56–58], and local region-based ACMs [34, 35, 59–62].

In particular, edge-based ACMs make use of an edge-detector (in general, the gradient of the image intensity) to try to stop the evolution of the active contour on the true boundaries of the objects of interest. One of the most popular edge-based active contours is the Geodesic Active Contour (GAC) model [24], which is described in the following.

*Geodesic Active Contour (GAC) Model [24].* The level set formulation of the GAC model can be described as follows:

(8) $$\begin{array}{c} \frac{\partial \phi }{\partial t}=g\left\|\nabla \phi \right\|\left(\nabla \cdot \left(\frac{\nabla \phi }{\left\|\nabla \phi \right\|}\right)+\alpha \right)+\nabla g\cdot \nabla \phi , \end{array}$$

where *ϕ* is the level set function, ∇ is the gradient operator, ∇· is the divergence operator, *α* > 0 is a “balloon” force term (controlling the rate of expansion of the level set function), and *g* is an Edge Stopping Function (ESF), defined as follows:

(9) $$\begin{array}{c} g∶=\frac{1}{1+{\left\|\nabla g_{\sigma }*I\right\|}^{2}}=\frac{1}{1+{\left\|g_{\sigma }*\nabla I\right\|}^{2}}, \end{array}$$

where *g*_*σ*_ is a Gaussian kernel function with width *σ*, ∗ is the convolution operator, and *I* is the image intensity. Hence, the ESF function provides information related to the gradient of the image intensity.

For images with a high level of noise, the presence of the Edge Stopping Function may not be enough to stop the contour evolution at the right boundaries. Motivated by this issue, a novel edge-based ACM has been proposed in [63] with the aim of improving the robustness of the segmentation to the noise. This has been achieved by regularizing the Laplacian of the image through an anisotropic diffusion term, which also preserves edge information.

Since edge-based models make use of an edge-detector to stop the evolution of the initial guess of the contour on the actual object boundaries, they can handle only images with well-defined edge information. Indeed, when images have ill-defined edges, the evolution of the contour typically does not converge to the true object boundaries.

An alternative solution consists in using statistical information about a region (e.g., intensity, texture, and color) to construct a stopping functional that is able to stop the contour evolution on the boundary between two different regions, as it happens in region-based models (see also the survey paper [64] for the recent state of the art of region-based ACMs) [26, 27]. An example of a region-based model is illustrated in the following.

*Chan-Vese (CV) Model [26].* The CV model is a well-known representative global region-based ACM (at the time of writing, it has received more than 4000 citations, according to Scopus). After its initialization, the contour in the CV model is evolved iteratively in an unsupervised fashion with the aim of minimizing a suitable energy functional, constructed in such a way that its minimum is achieved in correspondence with a close approximation of the actual boundary between two different regions. The energy functional *E*_CV_ of the CV model for a scalar-valued image has the expression

(10) ECVC∶=μ·LengthC+v·AreainC+λ+∫inCIx−c+C2dx+λ−∫out(C)Ix−c−C2dx,

where *C* is a contour, *I*(*x*) ∈ *ℝ* denotes the intensity of the image indexed by the pixel location *x* in the image domain *Ω*, *μ* ≥ 0 is a regularization parameter which controls the smoothness of the contour, in(*C*) (foreground) and out(*C*) (background) represent the regions inside and outside the contour, respectively, and *v* ≥ 0 is another regularization parameter, which penalizes a large area of the foreground. Finally, *c* ^+^(*C*) and *c* ^−^(*C*), which are defined, respectively, as

(11) $$\begin{array}{c} c^{+}\left(C\right)∶=\text{mean}\left(I\left(x\right)\;|\;x\in \text{in}\left(C\right)\right),\\ c^{-}\left(C\right)∶=\text{mean}\left(I\left(x\right)\;|\;x\in \text{out}\left(C\right)\right), \end{array}$$

represent the mean intensities of the foreground and the background, respectively, and *λ* ^+^, *λ* ^−^ ≥ 0 are parameters which control the influence of the two image energy terms ∫_in(*C*)_(*I*(*x*) − *c* ^+^(*C*))^2^ *dx* and ∫_out(*C*)_(*I*(*x*) − *c* ^−^(*C*))^2^ *dx*, respectively, inside and outside the contour. The functional is constructed in such a way that, when the regions in(*C*) and out(*C*) are smooth and “match” the true foreground and the true background, respectively, *E*_CV_(*C*) reaches its minimum.

Following [65], in the variational level set formulation of (10), the contour *C* is expressed as the zero level set of an auxiliary function *ϕ* : *Ω* → *ℝ*:

(12) $$\begin{array}{c} C∶=\left\{x\in \Omega :\phi \left(x\right)=0\right\}. \end{array}$$

Note that different functions *ϕ*(*x*) can be chosen to express the same contour *C*. For instance, denoting by *d*(*x*, *C*) the infimum of the Euclidean distances of the pixel *x* to the points on the curve *C*, *ϕ*(*x*) can be chosen as a signed distance function, defined as follows:

(13) $$\begin{array}{c} \phi \left(x\right)∶=\left\{\begin{array}{cc} d\left(x,C\right), & x\in \text{in}\left(C\right),\\ 0, & x\in C,\\ -d\left(x,C\right), & x\in \text{out}\left(C\right). \end{array}\right. \end{array}$$

This variational level set formulation has the advantage of being able to deal directly with the case of a foreground and a background that are not necessarily connected internally.

After replacing *C* with *ϕ* and highlighting the dependence of *c* ^+^(*C*) and *c* ^−^(*C*) on *ϕ*, in the variational level set formulation of the CV model the (local) minimization of the cost (10) is performed by applying the gradient-descent technique in an infinite-dimensional setting (see (7) and also the reference [26]), leading to the following PDE, which describes the evolution of the contour:

(14) ∂ϕ∂t=δϕμ∇·∇ϕ∇ϕ−v−λ+I−c+ϕ2   ∇ϕ∇ϕ+λ−I−c−(ϕ)2,

where *δ*(·) is the Dirac generalized function. The first term in *μ* of (14) keeps the level set function smooth, the second one in *v* controls the propagation speed of the evolving contour, while the third and fourth terms in *λ* ^+^ and *λ* ^−^ can be interpreted, respectively, as internal and external forces that drive the contour toward the actual object boundary. Then, (14) is solved iteratively in [26] by replacing the Dirac delta by a smooth approximation and using a finite difference scheme. Sometimes, also a reinitialization step is performed, in which the current level set function *ϕ* is replaced by its binarization (i.e., for a constant *ρ* > 0, a level set function of the form (3), representing the same current contour).

The CV model can also be derived, in a Maximum Likelihood setting, by making the assumption that the foreground and the background follow Gaussian intensity distributions with the same variance [21]. Then, the model approximates globally the foreground and background intensity distributions by the two scalars *c* ^+^(*ϕ*) and *c* ^−^(*ϕ*), respectively, which are their mean intensities. Similarly, Leventon et al. proposed in [66] to use Gaussian intensity distributions with different variances inside a parametric density estimation method. Also, Tsai et al. in [67] proposed to use instead uniform intensity distributions to model the two intensity distributions. However, such models are known to perform poorly in the case of objects with inhomogeneous intensities [21].

Compared to edge-based models, region-based models usually perform better in images with blurred edges and are less sensitive to the contour initialization.

Hybrid models that combine the advantages of both edge and regional information are able to control better the direction of evolution of the contour than the previously mentioned models. For instance, the Geodesic-Aided Chan-Vese (GACV) model [28] is a popular hybrid model, which includes both region and edge information in its formulation. Another example of a hybrid model is the following one.

*Selective Binary and Gaussian Filtering Regularized (SBGFRLS) Model [68].* The SBGFRLS model combines the advantages of both the CV and GAC models. It utilizes the statistical information inside and outside the contour to construct a region-based Signed Pressure Force (SPF) function, which is used in place of the edge stopping function (ESF) used in the GAC model (recall (9)). The SPF function is so called because it tends to make the contour *C* shrink when it is outside the object of interest and expand otherwise. The evolution of the contour in the variational level set formulation of the SBGFRLS model is described by the following PDE:

(15) $$\begin{array}{c} \frac{\partial \phi }{\partial t}=\text{spf}\left(I\left(x\right)\right)\cdot \alpha \left\|\nabla \phi \right\|, \end{array}$$

where *α* is a balloon force parameter and the SPF function spf is defined as

(16) $$\begin{array}{c} \text{spf}\left(I\left(x\right)\right)∶=\frac{I\left(x\right)-\left(\left(c^{+}\left(C\right)+c^{-}\left(C\right)\right)/2\right)}{\max_{x\in \Omega }\left(\left\|I\left(x\right)-\left(\left(c^{+}\left(C\right)+c^{-}\left(C\right)\right)/2\right)\right\|\right)}, \end{array}$$

where *c* ^+^(*C*) and *c* ^−^(*C*) are defined likewise in the CV model above. One can observe that, compared to the CV model, in (14) the Dirac delta term *δ*(*ϕ*) has been replaced by ‖∇*ϕ*‖ which, according to [68], has an effective range on the whole image, rather than the small range of the former. Also, the bracket in (14) is replaced by the function spf defined in (16). To regularize the curve *C*, the authors of [68] (following the practice consolidated in other papers, e.g., [22, 68, 69]), rather than relying on the computationally costly *μ*∇·(∇*ϕ*/‖∇*ϕ*‖) term, convolve the level set curve with a Gaussian kernel *g*_*σ*_; that is,

(17) $$\begin{array}{c} \phi ⟵g_{\sigma }*\phi , \end{array}$$

where the width *σ* of the Gaussian *g*_*σ*_ has a role similar to the one of *μ* in (14) of the CV model. If the value of *σ* is small, then the level set function is sensitive to the noise, and such a small value does not allow the level set function to flow into the narrow regions of the object to be segmented.

Overall this model is faster, is computationally more efficient, and performs better than the conventional CV model, as pointed out in [68]. However, it still has similar drawbacks as the CV model, such as its inefficiency in handling images with several intensity levels, its sensitivity to the contour initialization, and its inability to handle images with intensity inhomogeneity (arising, e.g., as an effect of slow variations in object illumination, possibly occurring during the image acquisition process).

In order to deal with images with intensity inhomogeneity, several authors have introduced in the SPF function terms that relate to local and global intensity information [34, 35, 59, 70]. However, these models are still sensitive to contour initialization and additive noise. Furthermore, when the contour is close to the object boundary, the influence of the global intensity force may distract the contour from the real object boundary, leading to object leaking [31], that is, the presence of a final blurred contour.

In general, global models cannot segment successfully objects that are constituted by more than one intensity class. On the other hand, sometimes this is possible by using local models, which rely on local information as their main component in the associated variational level set framework. However, such models are still sensitive to the contour initialization and may lead to object leaking. Some examples of such local region-based ACMs are illustrated in the following.

*Local Binary Fitting (LBF) Model [71].* The evolution of the contour in the LBF model is described by the following PDE:

(18) ∂ϕ∂t=−δεϕλ1e1−λ2e2+vδεϕ∇·∇ϕ∇ϕ+μ∇2ϕ−∇·∇ϕ∇ϕ,

where *v* and *μ* are nonnegative constants, ∇^2^ is the Laplacian operator, *ε* > 0, and the functions *e*_1_ and *e*_2_ are defined as follows:

(19) $$\begin{array}{c} e_{1}\left(x\right)∶=\int_{\Omega }g_{\sigma }\left(x-y\right){\left(I\left(y\right)-f_{1}\left(x\right)\right)}^{2}dy,\\ e_{2}\left(x\right)∶=\int_{\Omega }g_{\sigma }\left(x-y\right){\left(I\left(y\right)-f_{2}\left(x\right)\right)}^{2}dy, \end{array}$$

where *f*_1_ and *f*_2_ are, respectively, internal and external gray-level fitting functions, and *g*_*σ*_(*x*) is a Gaussian kernel function of width *σ*. Also, for *ε* > 0, *δ*_*ε*_(*ϕ*) is a suitable regularized Dirac delta function, defined as follows:

(20) $$\begin{array}{c} \delta_{\varepsilon }\left(x\right)=\frac{1}{\pi }\frac{\varepsilon }{\varepsilon^{2}+x^{2}}. \end{array}$$

In more details, the functions *f*_1_ and *f*_2_ are defined as

(21) $$\begin{array}{c} f_{1}\left(x\right)∶=\frac{g_{\sigma }\left(x\right)\left[H\left(\phi \left(x\right)\right)I\left(x\right)\right]}{g_{\sigma }\left(x\right)H\left(\phi \left(x\right)\right)},\\ f_{2}\left(x\right)∶=\frac{g_{\sigma }\left(x\right)\left[\left(1-H\left(\phi \left(x\right)\right)\right)I\left(x\right)\right]}{g_{\sigma }\left(x\right)\left(1-H\left(\phi \left(x\right)\right)\right)}. \end{array}$$

In general, the LBF model can produce good segmentations of objects with intensity inhomogeneities. Furthermore, it has a better performance than the well-known Piecewise Smooth (PS) model [33, 72] for what concerns segmentation accuracy and computational efficiency. However, the LBF model only takes into account the local gray-level information. Thus, in this model, it is easy to be trapped into a local minimum of the energy functional, and the model is also sensitive to the initial location of the active contour. Finally, oversegmentation problems may occur.

*Local Image Fitting (LIF) Energy Model [32].* Zhang et al. proposed in [32] the LIF energy model to insert local image information in their energy functional. The evolution of the contour in the LIF model is described by the following PDE:

(22) $$\begin{array}{c} \frac{\partial \phi }{\partial t}=\left(I-I^{\text{LFI}}\right)\left(m_{1}-m_{2}\right)\delta_{\varepsilon }\left(\phi \right), \end{array}$$

where the intensity *I* ^LFI^ of the local fitted image LFI is defined as follows:

(23) $$\begin{array}{c} I^{\text{LFI}}∶=m_{1}H_{\varepsilon }\left(\phi \right)+m_{2}\left(1-H_{\varepsilon }\left(\phi \right)\right), \end{array}$$

where *m*_1_ and *m*_2_ are the average local intensities inside and outside the contour, respectively.

The main idea of this model is to use the local image information to construct an energy functional, which takes into account the difference between the fitted image and the original one to segment an image with intensity inhomogeneities. The complexity analysis and experimental results showed that the LIF model is more efficient than the LBF model, while yielding similar results.

However, the models above are still sensitive to the contour initialization, and to high levels of additive noise. Compared to the two above-mentioned models, a model that has shown higher accuracy when handling images with intensity inhomogeneity is the following one.

*Local Region-Based Chan-Vese (LRCV) Model [31].* The LRCV model is a natural extension of the already-mentioned Chan-Vese (CV) model. Such an extension is obtained by inserting local intensity information into the objective functional. This is the main feature of the LRCV model, which provides to it the capability of handling images with intensity inhomogeneity, which is missing instead in the CV model.

The objective functional *E*_LRCV_ of the LRCV model has the expression

(24) ELRCVC∶=λ+∫inCIx−c+x,C2dx+λ−∫out(C)Ix−c−x,C2dx,

where *c* ^+^(*x*, *C*) and *c* ^−^(*x*, *C*) are functions which represent the local weighted mean intensities of the image around the pixel *x*, assuming that it belongs, respectively, to the foreground/background:

(25) $$\begin{array}{c} c^{+}\left(x,C\right)∶=\frac{\int_{\text{in}(C)}g_{\sigma }\left(x-y\right)I\left(y\right)dy}{\int_{\text{in}(C)}g_{\sigma }\left(x-y\right)dy},\\ c^{-}\left(x,C\right)∶=\frac{\int_{\text{out}(C)}g_{\sigma }\left(x-y\right)I\left(y\right)dy}{\int_{\text{out}(C)}g_{\sigma }\left(x-y\right)dy}, \end{array}$$

where *g*_*σ*_ is a Gaussian kernel function with ∫_*ℝ*^2^_ *g*_*σ*_(*x*)*dx* = 1 and width *σ* > 0.

The evolution of the contour in the LRCV model is described by the following PDE:

(26) $$\begin{array}{c} \frac{\partial \phi }{\partial t}=\delta \left(\phi \right)\left[-\lambda^{+}{\left(I-c^{+}(x,\phi )\right)}^{2}+\lambda^{-}{\left(I-c^{-}(x,\phi )\right)}^{2}\right]. \end{array}$$

Equation (26) can be solved iteratively by replacing the Dirac delta by a smooth approximation and using a finite difference scheme. Moreover, one can perform also a regularization step, in which the current level set function *ϕ* is replaced by its convolution by a Gaussian kernel with suitable width *σ*′ > 0.

A drawback of the LRCV model is that it relies only on the local information coming from the current location of the contour, so it is sensitive to the contour initialization.

*Locally Statistical Active Contour Model (LSACM) [73].* This model has been proposed with the aim of handling images characterized by intensity inhomogeneity, and of being robust to the contour initialization. It can be considered as a generalization of the Local Intensity Clustering (LIC) model proposed in [74], which is applicable for both simultaneous segmentation and bias correction.

The evolution of the level set function in the LSACM model is controlled by the following gradient descent formulation:

(27) $$\begin{array}{c} \frac{\partial \phi }{\partial t}=\left(d_{2}-d_{1}\right)\delta \left(\phi \right), \end{array}$$

where *d*_1_ and *d*_2_ are two functions (one related to the foreground, the other one to the background), having suitable integral representations. Due to this fact, the LSACM model is able to combine the information about the spatial dependencies between pixels belonging to the same class and yields a soft segmentation. However, like the previous model, also this one is characterized by a high computational cost, in addition to the limitation of relying on a particular probabilistic model.

### 2.2. Supervised Models

From a machine learning perspective, ACMs for image segmentation can use both supervised and unsupervised information. Both kinds of ACMs rely on parametric and/or nonparametric density estimation methods to approximate the intensity distributions of the subsets to be segmented (e.g., foreground/background). Often, in such models one makes statistical assumptions on the image intensity distribution, and the segmentation problem is solved by a Maximum Likelihood (ML) or Maximum A-Posteriori (MAP) probability approach. For instance, for scalar-valued images, in both parametric/nonparametric region-based ACMs, the objective energy functional has usually an integral form (see, e.g., [75]), whose integrands are expressed in terms of functions *e*_*i*_(*x*) having the form:

(28) $$\begin{array}{c} e_{i}\left(x\right)∶=-\log\left(p_{i}\left(I\left(x\right)\right)\right),\;\forall i\in I, \end{array}$$

where *ℐ* is the number of objects (subsets) *Ω*_*i*_ to be segmented. Here, *p*_*i*_(*I*(*x*))∶ = *p*(*I*(*x*)∣*x* ∈ *Ω*_*i*_) is the conditional probability density of the image intensity *I*(*x*), conditioned on *x* ∈ *Ω*_*i*_, so the log-likelihood term log⁡  (*p*_*i*_(*I*(*x*))) quantifies how much an image pixel is likely to be an element of the subset *Ω*_*i*_. In the case of supervised ACMs, the models *p*_*i*_(*I*(*x*)) are estimated from a training set, one for each subset *Ω*_*i*_. Similarly, for a vector-valued image **I**(*x*) with *D* components, the terms *e*_*i*_(*x*) have the form:

(29) $$\begin{array}{c} e_{i}\left(x\right)∶=-\log\left(p_{i}\left(I\left(x\right)\right)\right),\;\forall i\in I, \end{array}$$

where *p*_*i*_(**I**(*x*))∶ = *p*(**I**(*x*)∣*x* ∈ *Ω*_*i*_).

Now, we briefly discuss some supervised ACMs, which take advantage of the availability of labeled training data. As an example, Lee et al. proposed in [75] a supervised ACM, which is formulated in a parametric form. In the following, we refer to such a model as a Gaussian Mixture Model (GMM-) based ACM, since it exploits supervised training examples to estimate the parameters of multivariate Gaussian mixture densities. In such a model, the level set evolution PDE is given, for example, in the case of multispectral images **I**(*x*), by

(30) $$\begin{array}{c} \frac{\partial \phi }{\partial t}=\delta \left(\phi \right)\left[\beta \kappa \left(\phi \right)+\log\;p_{\text{in}}\left(I\right)-\log\;p_{\text{out}}\left(I\right)\right], \end{array}$$

where *β* ≥ 0 is a regularization parameter and *κ*(*ϕ*) is the average curvature of the level set function *ϕ*.

The two terms *p*_in_(**I**(*x*)) and *p*_out_(**I**(*x*)) in (30) are then expressed in [75] as

(31) $$\begin{array}{c} p_{\text{in}}\left(I\left(x\right)\right),p_{\text{out}}\left(I\left(x\right)\right)∶=\overset{K}{\underset{k=1}{\sum }}\alpha_{k}N\left(\mu_{k},\Sigma_{k},I\left(x\right)\right), \end{array}$$

where *K* is the number of computational units, *𝒩*(*μ*_*k*_, Σ_*k*_, ·), *k* = 1,…, *K* are Gaussian functions with centers *μ*_*k*_ and covariance matrices Σ_*k*_, and the *α*_*k*_'s are the coefficients of the linear combination. All the parameters (*α*_*k*_, *μ*_*k*_, Σ_*k*_) are then estimated from the training examples. Besides GMM-based ACMs, also nonparametric Kernel Density Estimation (KDE-) based models with Gaussian computational units have been proposed in [76, 77] with the same aim. In the case of scalar images, they have the form:

(32) $$\begin{array}{c} p_{\text{in}}\left(I\left(x\right)\right)∶=\frac{1}{\left|L^{+}\right|}\overset{\left|L^{+}\right|}{\underset{i=1}{\sum }}K\left(\frac{I\left(x\right)-I\left(x_{i}^{+}\right)}{\sigma_{\text{KDE}}}\right),\\ p_{\text{out}}\left(I\left(x\right)\right)∶=\frac{1}{\left|L^{-}\right|}\overset{\left|L^{-}\right|}{\underset{i=1}{\sum }}K\left(\frac{I\left(x\right)-I\left(x_{i}^{-}\right)}{\sigma_{\text{KDE}}}\right), \end{array}$$

where the pixels *x*_*i*_^+^ and *x*_*i*_^−^ belong, respectively, to given sets *L* ^+^ and *L* ^−^ of training pixels inside the true foreground/background (with cardinalities |*L* ^+^| and |*L* ^−^|, resp.), *σ*_KDE_ > 0 is the width of the Gaussian kernel used in the KDE-based model, and

(33) $$\begin{array}{c} K\left(u\right)∶=\frac{1}{\sqrt{2\pi }}\exp\left(-\frac{u^{2}}{2}\right). \end{array}$$

Of course, such models can be extended to the case of vector-valued images (in particular, replacing *σ*_KDE_^2^ by a covariance matrix).

### 2.3. Other Variational Level Set-Based ACMs

*Supervised Boundary-Based GAC (sBGAC) Model [78].* The sBGAC model is a supervised level set-based ACM, which was proposed by Paragios and Deriche with the aim of providing a boundary-based framework that is derived by the GAC for texture image segmentation. Its main contribution is the connection between the minimization of a GAC objective with a contour propagation method for supervised texture segmentation. However, sBGAC is still limited to boundary-based information, which results in a high sensitivity to the noise and to the initial contour.

*Geodesic Active Region (GARM) Model [79].* GARM was proposed with the aim of reducing the sensitivity of sBGAC to the noise and to the contour initialization, by integrating the region-based information along with the boundary information. GARM is a supervised texture segmentation ACM implemented by a variational level set method.

The inclusion of supervised examples in ACMs can improve significantly their performance by constructing a Knowledge Base (KB), to be used as a guide in the evolution of the contour. However, state-of-the-art supervised ACMs often make strong statistical assumptions on the image intensity distribution of each subset to be modeled. So, the evolution of the contour is driven by probability models constructed based on given reference distributions. Therefore, the applicability of such models is limited by how accurate the probability models are.

## 3. SOM-Based ACMs

Before discussing SOM-based ACMs, we shortly review the use of SOMs as a tool in pattern recognition (hence, in image segmentation as a particular case).

### 3.1. Self-Organizing Maps (SOMs)

The SOM [37], which was proposed by Kohonen, is an unsupervised neural network whose neurons update concurrently their weights in a self-organizing manner, in such a way that, during the learning process, the weights of the neurons evolve adaptively into specific detectors of different input patterns. A basic SOM is composed of an input layer, an output layer, and an intermediate connection layer. The input layer contains a unit for each component of the input vector. The output layer consists of neurons that are typically located either on a 1-*D* or a 2-*D* grid and are fully connected with the units in the input layer. The intermediate connection layer is composed of weights (also called prototypes) connecting the units in the input layer and the neurons in the output layer (in practice, one has one weight vector associated with each output neuron, where the dimension of the weight vector is equal to the dimension of the input). The learning algorithm of the SOM can be summarized by the following steps:

1. Initialize randomly the weights of the neurons in the output layer, and select suitable learning rate and neighborhood size around a “winner” neuron.
2. For each training input vector, find the winner neuron, also called Best Matching Unit (BMU) neuron, using a suitable rule.
3. Update the weights on the selected neighborhood of the winner neuron.
4. Repeat Steps (2)-(3) above selecting another training input vector, until learning is accomplished (i.e., a suitable stopping criterion is satisfied).

More precisely, after its random initialization, the weight *w*_*j*_ of each neuron *j* is updated at each iteration *t* through the following self-organization learning rule:

(34) $$\begin{array}{c} w_{j}\left(t+1\right)∶=w_{j}\left(t\right)+\eta \left(t\right)h_{j,b}\left(t\right)\left(x^{\left(t\right)}-w_{j}\left(t\right)\right), \end{array}$$

where *x* ^(*t*)^ is the input of the SOM at time *t*, *η*(*t*) is a learning rate, and *h*_*j*,*b*_(*t*) is a neighborhood kernel around the BMU neuron *b* (i.e., the neuron whose weight vector *w*_*b*_ is the closest to the input *x* ^(*t*)^). Both functions *η*(*t*) and *h*_bn_(*t*) are designed to be time-decreasing in order to stabilize the weights *w*_*j*_(*t*) for *t* sufficiently large. Usual choices of the functions above are

(35) $$\begin{array}{c} \eta \left(t\right)∶=\eta_{0}\exp\left(-\frac{t}{\tau_{\eta }}\right), \end{array}$$

where *η*_0_ > 0 is the initial learning rate and *τ*_*η*_ > 0 is a time constant, and

(36) $$\begin{array}{c} h_{j,b}\left(t\right)∶=\exp\left(-\left(\frac{d_{j,b}^{2}}{\sigma_{b}^{2}\left(t\right)}\right)\right), \end{array}$$

where *d*_*j*,*b*_ is the distance between the neurons *j* and *b*, and *σ*_*b*_(*t*) is a suitable choice for the width of the Gaussian function in (36).

SOMs have been used extensively for image segmentation, but often not in combination with ACMs [80, 81]. In the following subsection, we review, in brief, some of the existing SOM-based segmentation models which are not related to ACMs.

#### 3.1.1. SOM-Based Segmentation Models Not Related to ACMs

In [82], a SOM-based clustering technique was used as a thresholding technique for image segmentation. The idea was to apply the intensity histogram of the image to feed a SOM that divides the histogram into regions. Huang et al. in [83] proposed to use a two-stages SOM system in segmenting multispectral images (specifically, made of three components, or channels). In the first stage, the goal was to identify a large initial set of color classes, while the second stage aimed to identify a final batch of segmented clusters. In [84], Jiang et al. used SOMs to segment multispectral images (specifically, made of five components), by clustering the pixels based on their color and on other spatial features. Then, those clustered regions were merged into a predefined number of regions by the application of some morphological operations. Concluding, SOMs have been extensively used in the field of segmentation, and, as stated in [85–90], the SOM-based segmentation models proposed in the literature yielded improved segmentation results compared to the direct application of the classical SOM.

Although SOMs are traditionally associated with unsupervised learning, in the literature there exist also supervised SOMs. A representative model of a supervised SOM is the Concurrent Self-Organizing Map (CSOM) [91], which combines several SOMs to deal with the pattern classification problem (hence, the image segmentation problem as a particular case) in a parallel processing way, with the aim of minimizing a suitable objective function, usually the quantization error of the maps. In a CSOM, each SOM is constructed and trained individually on a subset of examples coming only from its associated class. The aim of this training is to increase the discriminative capability of the system. So, the training of the CSOM is supervised for what concerns the assigment of the training examples to the various SOMs, but each individual SOM is trained with the SOM specific self-organizing (hence, unsupervised) learning rule.

We conclude mentioning that, when SOMs are used as supervised/unsupervised image segmentation techniques, the application of the resulting model usually produces segmented objects characterized by disconnected boundaries, and the segmentation result is often sensitive to the noise.

#### 3.1.2. SOM-Based Segmentation Models Related to ACMs

In order to improve the robustness of edge-based ACMs to the blur and to ill-defined edge information, SOMs have been also used in combination with ACMs, with the explicit aim of modelling the active contour and controlling its evolution, adopting a learning scheme similar to Kohonen's learning algorithm [37], resulting in SOM-based ACMs [38, 39] (which belong, in the case of [38, 39], to the class of edge-based ACMs). The evolution of the active contour in a SOM-based ACM is guided by the feature space constructed by the SOM when learning the weights associated with the neurons of the map. Moreover, other kinds of neural networks have been used with the aim of approximating the edge map: for example, multilayer perceptrons [92]. One reason to prefer SOMs to other neural network models consists in the specific ability of SOMs to learn, for example, the edge-map information via their topology preservation property. A review of SOM-based ACMs belonging to the class of edge-based ACMs is provided in the two following subsections, whereas Section 4 presents a more recent class of SOM-based ACMs combined with variational level set methods.

### 3.2. An Example of a SOM-Based ACM Belonging to the Class of Edge-Based ACMs

The basic idea of existing SOM-based ACMs belonging to the class of edge-based ACMs is to model and implement the active contour using a SOM, relying in the training phase on the edge map of the image to update the weights of the neurons of the SOM, and consequently to control the evolution of the active contour. The points of the edge map act as inputs to the network, which is trained in an unsupervised way (in the sense that no supervised examples belonging to the foreground/background, resp., are provided). As a result, during training the weights associated with the neurons in the output map move toward points belonging to the nearest salient contour. In the following, we illustrate the general ideas of using a SOM in modelling the active contour, by describing a classical example of a SOM-based ACM belonging to the class of edge-based ACMs, which was proposed in [38] by Venkatesh and Rishikesh. 

*Spatial Isomorphism Self-Organizing Map (SISOM-) Based ACM [38].* This is the first SOM-based ACM which appeared in the literature. It was proposed with the aim of localizing the salient contours in an image using a SOM to model the evolving contour. The SOM is composed of a fixed number of neurons (and consequently a fixed number of “knots” or control points for the evolving curve) and has a fixed structure. The model requires a rough approximation of the true boundary as an initial contour. Its SOM network is constructed and trained in an unsupervised way, based on the initial contour and the edge-map information. The contour evolution is controlled by the edge information extracted from the image by an edge detector. The main steps of the SISOM-based ACM can be summarized as follows:

1. Construct the edge map of the image to be segmented.
2. Initialize the contour to enclose the object of interest in the image.
3. Obtain the horizontal and vertical coordinates of the edge points to be presented as inputs to the network.
4. Construct a SOM with a number of neurons equal to the number of the edge points of the initial contour and two scalar weights associated with each neuron; the points on the initial contour are used to initialize the SOM weights.
5. Repeat the following steps for a fixed number of iterations:
  - Select randomly an edge point and feed its coordinates to the network.
  - Determine the best-matching neuron.
  - Update the weights of the neurons in the network by the classical unsupervised learning scheme of the SOM [37], which is composed of a competitive phase and a cooperative one.
  - Compute a neighborhood parameter for the contour according to the updated weights and a threshold.

**Figure 3:**
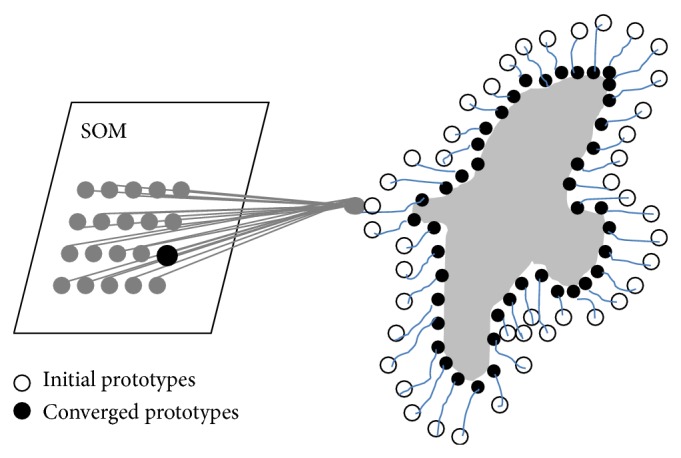
The architecture of the SISOM-based ACM proposed in [38].

Figure 3 illustrates the evolution procedure of the SISOM-based ACM. On the left-side of the figure, the neurons of the map are represented by gray circles, while the black circle represents the winner neuron associated with the current input to the map (in this case, the gray circle on the right-hand side of the figure, which is connected by the gray segments to all the neurons of the map). On the right-hand side, instead, the positions of the white circles represent the initial prototypes of the neurons, whereas the positions of the black circles represent their final values, at the end of learning. The evolution of the contour is controlled by the learning algorithm above, which guides the evolution of the prototypes of the neurons of the SOM (hence, of the active contour) using the points of the edge map as inputs to the SOM learning algorithm. As a result, the final contour is represented by a series of prototypes of neurons located near the actual boundary of the object to be segmented.

We conclude by mentioning that, in order to produce good segmentations, the SISOM-based ACM requires the initial contour (which is used to initialize the prototypes of the neurons) to be very close to the true boundary of the object to be extracted, and the points of the initial contour have to be assigned to the neurons of the SOM in a suitable order: if such assumptions are satisfied, then the contour extraction process performed by the model is generally robust to the noise. Moreover, differently from other ACMs, this model does not require a particular energy functional to be optimized.

### 3.3. Other SOM-Based ACMs Belonging to the Class of Edge-Based ACMs

In this subsection, we describe other SOM-based ACMs belonging also to the class of edge-based ACMs, and highlight their advantages and disadvantages. 

*Time Adaptive Self-Organizing Map (TASOM-) Based ACM [39].* The TASOM-based ACM was proposed by Shah-Hosseini and Safabakhsh as a development of the SISOM-based ACM, with the aim of inserting neurons incrementally into the SOM map or deleting them incrementally, thus determining automatically the required number of control points of the extracted contour. The addition and deletion processes are based on the closeness of any two adjacent neurons *j* and *j* + 1. More precisely, if the distance *d*(*w*_*j*_, *w*_*j*+1_) between the corresponding weights *w*_*j*_ and *w*_*j*+1_ is smaller than a given threshold *θ*_*l*_ > 0, then the two neurons are merged, whereas a new neuron is inserted between the two neurons if *d*(*w*_*j*_, *w*_*j*+1_) is larger than another given threshold *θ*_*h*_ > 0. Moreover, at each time *t*, each neuron *j* is provided with its specific dynamic learning rate *η*_*j*_(*t*), which is defined as follows:

(37) $$\begin{array}{c} \eta_{j}\left(t+1\right)∶=\left(1-\alpha \right)\eta_{j}\left(t\right)+\alpha f\left(\frac{\left\|x^{\left(t\right)}-w_{j}\left(t\right)\right\|}{s_{f}\cdot sl\left(t\right)}\right), \end{array}$$

where *α* ∈ (0,1) is a constant, *s*_*f*_ is a positive constant which controls the slope of *f*, *x* ^(*t*)^ is the input at time *t*, and *sl*(*t*) is a suitable scaling function, which makes the SOM network invariant to scaling transformations. Finally, at each time *t*, each neuron *j* is also associated with the neighborhood function *h*_*j*,*b*_(*t*), which has the form of (36).

The TASOM-based ACM can overcome one of the main limitations of the SISOM-based ACM, that is, its sensitivity to the contour initialization, in the sense that, for a successful segmentation, the initial guess of the contour in the TASOM-based ACM can be even far from the actual object boundary. Likewise in the case of the SISOM-based ACM, topological changes of the objects (e.g., splitting and merging) cannot be handled by the TASOM-based ACM, since both models rely completely on the edge information (instead than on regional information) to drive the contour evolution. 

*Batch Self-Organizing Map (BSOM-) Based ACM [20, 93].* This model is a modification of the TASOM-based ACM, and was proposed by Venkatesh et al. with the aim of dealing better with the leaking problem (i.e., the presence of a final blurred contour), which often occurs when handling images with ill-defined edges. Such a problem is due to the explicit use by the TASOM-based ACM of only edge information to model and control the evolution of the contour. The BSOM-based ACM, instead, relies on the fact that the image intensity variation inside a local region can be used in a way to increase the robustness of the model during the movements of the contour. As a consequence, the BSOM-based ACM associates a region boundary term *u*(*j*) with each neuron *j*, in order to control better the movements of the neurons. Such a term is defined as

(38) $$\begin{array}{c} u\left(j\right)∶=\overset{N_{r}}{\underset{k=1}{\sum }}\mathrm{sgn}\left(I\left(p_{a_{k}}\right)-I\left(p_{b_{k}}\right)\right), \end{array}$$

where *N*_*r*_ is the number of neighborhood points of the neuron *j* that are taken into account for the local analysis of the region boundary, *I*(·) is the image intensity function, sgn is the signum function, and *p*_*a*_*k*__, *p*_*b*_*k*__ are suitable neighborhood points of the neuron *j*, outside and inside the contour, respectively. Now, the sign of the difference in (38) between the image intensities at the points *p*_*a*_*k*__ and *p*_*b*_*k*__ should be the same for all *k*, if the neuron *j* is near a true region boundary. In this way, the robustness of the model is increased in handling images with blurred edges. At the same time, the BSOM-based ACM is less sensitive to the initial guess of the contour, when compared to parametric ACMs like Snakes, and to the SOM-based ACMs described above. However, like all such models, the BSOM-based ACM has not the ability to handle topological changes of the objects to be segmented. An extension of the BSOM-based ACM was proposed in [94, 95] and applied therein to the segmentation of pupil images. Such a modified version of the basic BSOM-based ACM increases the smoothness of the extracted contour, and prevents the extracted contour from being extended over the true boundaries of the object. 

*Fast Time Adaptive Self-Organizing Map (FTA-SOM-) Based ACM [96].* This is another modification of the TASOM-based ACM, and it was proposed by Izadi and Safabakhsh with the aim of decreasing the computational complexity of the method, by using an adaptive speed parameter instead of the one used in [39], which was fixed, instead. Such an adaptive speed parameter was also proposed with the aim of increasing the speed of convergence and accuracy. The FTA-SOM-based ACM is also based on the observation that choosing the learning rate parameters of the prototypes of the neurons of the SOM in such a way that they are equal to a large fixed value when they are far from the boundary, and to a small value when they are near the boundary, can lead to a significant increase of the convergence speed of the active contour. Accordingly, in each iteration, the FTA-SOM-based ACM finds the minimum distance of each neuron from the boundary, then it sets the associated learning rate as a fraction of that distance. 

*Coarse to Fine Boundary Location Self-Organizing Map (CFBL-SOM-) Based ACM [97].* The above SOM-based ACMs work in an unsupervised way, as the user is required only to provide an initial contour to be evolved automatically. In [97], Zeng et al. proposed the CFBL-SOM-based ACM as the first supervised SOM-based ACM, that is, a model in which the user is allowed to provide supervised points (supervised “seeds”) from the desired boundaries. Starting from this coarse information, the SOM neurons are then employed to evolve the contour to the desired boundaries in a “coarse-to-fine” approach. The CFBL-SOM-based ACM follows such a strategy, when controlling the evolution of the contour. So, an advantage of the CFBL-SOM-based ACM over the SOM-based ACMs described above is that it allows to integrate prior knowledge about the desired boundaries of the objects to be segmented, which comes from the user interaction with the SOM-based ACM segmentation framework. When compared to such SOM-based ACMs, this property provides the CFBL-SOM-based ACM with the ability of handling objects with more complex shapes, inhomogeneous intensity distributions, and weak boundaries.

**Figure 4:**
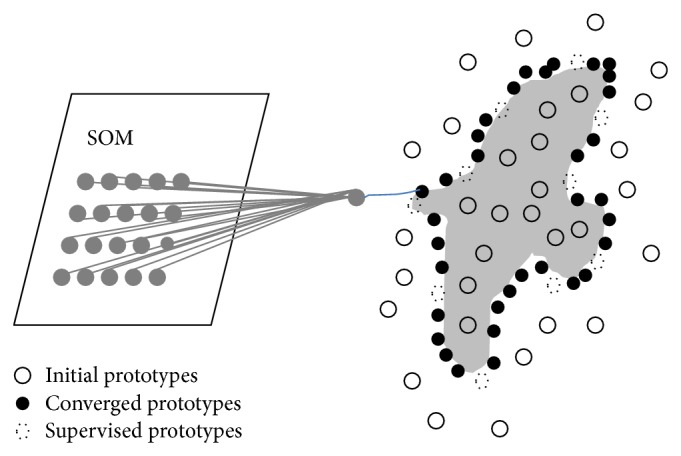
The architecture of the CFBL-SOM-based ACM proposed in [97].

Figure 4 illustrates the evolution procedure of the CFBL-SOM-based ACM, which is similar to the one of the SISOM-based ACM. The only difference is represented by the dashed circles, which are used as supervised pixels to increase the robustness of the model to the initialization of the contour. Due to this reason, for a successful segmentation, the white circles in Figure 4 can be initialized even far away from the actual boundary of the object, differently from Figure 3. Finally, due to the presence of the supervision, this method also allows one to handle more complex images.

*Conscience, Archiving and Mean-Movement Mechanisms Self-Organizing Map (CAM-SOM-) Based ACM [98].* The CAM-SOM-based ACM was proposed by Sadeghi et al. as an extension of the BSOM-ACM, by introducing three mechanisms called Conscience, Archiving and Mean-movement. The main achievement of the CAM-SOM-based ACM is to allow more complex boundaries (such as concave boundaries) to be captured, and to provide a reduction of the computational cost. By the Conscience mechanism, the neurons are not allowed to “win” too much frequently, which makes the capture of more complex boundaries possible. The Archiving mechanism allows a significant reduction in the computational cost. By such mechanism, neurons whose prototypes are close to the boundary of the object to be segmented and whose values have not changed significantly in the last iterations are archived and eliminated from subsequent computations. Finally, in order to ensure a continuous movement of the active contour toward concave regions, the Mean-movement mechanism is used in each epoch to force the winner neuron to move toward the mean of a set of feature points, instead of a single feature point. Together, the Conscience and Mean-movement mechanisms prevent the contour from stopping the contour evolution at the entrance of object concavities. 

*Extracting Multiple Objects.* The main limitation of various of the SOM-based ACMs reviewed above is their inability to detect multiple contours and to recognize multiple objects. A similar problem arises in parametric ACMs such as Snakes. To deal with the multiple contour extraction problem, Venkatesh et al. proposed in [93] to use a splitting criterion. However, if the initial contour is outside the objects, contours inside an object still cannot be extracted by using such a criterion. Sadeghi et al. proposed in [98] another splitting criterion (to be checked at each epoch) such that the main contour can be divided into several subcontours whenever the criterion is satisfied. The process is repeated until each of the subcontours encloses one single object. However, the merging process is still not handled implicitly by the model, which reduces its scope, especially when handling images containing multiple objects in the presence of noise or ill-defined edges. Moreover, Ma et al. proposed in [99] to use a SOM to classify the edge elements in the image. This model relies first on detecting the boundaries of the objects. Then, for each edge pixel, a feature vector is extracted and normalized. Finally, a SOM is used as a clustering tool to detect the object boundaries when the feature vectors are supplied as inputs to the map. As a result, multiple contours can be recognized. However, the model shares the same limitations of other models that use a SOM as a clustering tool for image segmentation [80, 100, 101], resulting in disconnected boundaries and a high sensitivity to the presence of the noise.

## 4. SOM-Based ACMs Combined with Variational Level Set Methods

Recently, a new class of SOM-based ACMs combined with variational level set methods has been proposed in [102–105], with the aim of taking advantage of both SOMs and variational level set methods, in order to handle images presenting challenges in computer vision in an efficient, effective, and robust way. In this section, we describe the main contributions of such approaches, by comparing them with the above-mentioned active contour models.

*Concurrent Self-Organizing Map-Based Chan-Vese (CSOM-CV) Model.* CSOM-CV [102] is a novel regional ACM, which relies on a CSOM made of two SOMs to approximate the foreground and background image intensity distributions in a supervised fashion, and to drive the evolution of the active contour accordingly. The model integrates such an information inside the framework of the Chan-Vese (CV) model, hence the name of such a model is Concurrent Self-Organizing Map-based Chan-Vese (CSOM-CV) model. The main idea of the CSOM-CV model is to concurrently integrate the global information extracted by a CSOM from a few supervised pixels into the level-set framework of the CV model to build an effective ACM. The proposed model integrates the advantages of the CSOM as a powerful classification tool and of the CV model as an effective tool for the optimization of a global energy functional. The evolution of the contour in the CSOM-CV model (which is a variational level set method) is described by the following PDE:

(39) $$\begin{array}{c} \frac{\partial \phi }{\partial t}=\delta \left(\phi \right)\left[-\lambda^{+}e^{+}+\lambda^{-}e^{-}\right], \end{array}$$

where *e* ^+^(*x*, *C*) and *e* ^−^(*x*, *C*) are two energy terms, which are used to determine the forces acting inside and outside the contour, respectively. They are defined, respectively, as

(40) $$\begin{array}{c} e^{+}\left(x,C\right)∶={\left(I(x)-w_{b}^{+}(C)\right)}^{2}, \end{array}$$

(41) $$\begin{array}{c} e^{-}\left(x,C\right)∶={\left(I(x)-w_{b}^{-}(C)\right)}^{2}, \end{array}$$

where *w*_*b*_^+^(*C*) is the prototype of the neuron of the first SOM that is the BMU neuron to the mean intensity inside the current contour, while *w*_*b*_^−^(*C*) is the prototype of the neuron of the second SOM that is the BMU neuron to the mean intensity outside it.

**Figure 5:**
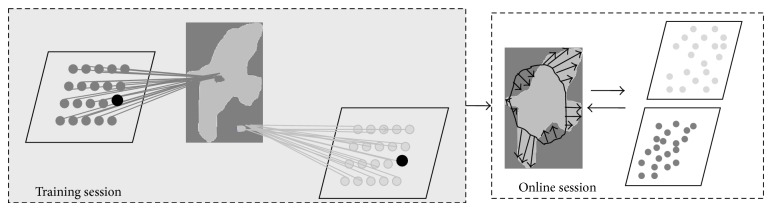
The architecture of the CSOM-CV ACM proposed in [102].

Figure 5 illustrates the off-line (i.e., training session) and on-line components of the CSOM-CV model. In the off-line session, the foreground supervised pixels are represented in light gray, while the background ones are represented in dark gray. The first SOM is trained using the intensity of the foreground supervised pixels, whereas the second one is trained using the intensity of the background supervised pixels. In such a session, the neurons of the two SOMs are arranged in such a way that the topological structure of the foreground and background intensity distributions are preserved. Finally, in the online session, the learned prototypes of the “foreground” and “background” neurons associated, respectively, with the two SOMs (and represented in light and dark gray, resp., in Figure 5) are used implicitly to control the evolution of the contour toward the true object boundary.

*Self-Organizing Active Contour (SOAC) Model [103].* Likewise the CSOM-CV model, also the SOAC model combines a variational level set method with the prototypes associated with the neurons of a SOM, which are learned during the off-line phase. Then, in the online phase, the contour evolution is implicitly controlled by the minimization of the quantization error of the organized neurons. The SOAC model can handle images with multiple intensity classes, intensity inhomogeneity, and complex distributions with a complicated foreground and background overlap. Compared to CSOM-CV, the SOAC model makes the following important improvement: its regional descriptors *w*_*b*_^+^(*x*, *C*) and *w*_*b*_^−^(*x*, *C*) (which are used in a similar way as the ones *w*_*b*_^+^(*C*) and *w*_*b*_^−^(*C*) in (4) and (41), resp.) depend on the pixel location *x*, while CSOM-CV uses the regional descriptors *w*_*b*_^+^(*C*) and *w*_*b*_^−^(*C*), which are constant functions. So, CSOM-CV is a global ACM (i.e., the spatial dependencies of the pixels are not taken into account in such a model, since it just considers only the average intensities inside and outside the contour), whereas the SOAC model makes also use of local information, which provides it the ability of handling more complex images. Finally, the experimental results reported in [102] have shown the higher accuracy of the segmentation results obtained by SOAC on several synthetic and real images compared to some well-known ACMs. 

*SOM-Based Chan-Vese (SOMCV) Model [104]. *This is similar to the CSOM-CV model, with the difference that now the training of the model is completely unsupervised, differently from the two previous models. Likewise for the CSOM-CV model, the prototypes of the trained neurons encode global intensity information also in this case. The SOMCV model can handle images with many intensity levels and complex intensity distributions, and it is robust to additive noise. Experimental results reported in [104] have shown the higher accuracy of the segmentation results obtained by the SOMCV model on several synthetic and real images, when compared to the CV active contour model. A significant difference with the CSOM-CV model is that the intervention of the final user is significantly reduced in the SOMCV model, since no supervised information is used. Finally, SOMCV has a Self-Organizing Topology Preservation (SOTP) property, which allows to preserve the topological structures of the foreground/background intensity distributions during the active contour evolution. Indeed, SOMCV relies on a set of self-organized neurons by automatically extracting the prototypes of selected neurons as global regional descriptors and iteratively, in an unsupervised way, integrates them in the evolution of the contour. 

*SOM-Based Regional Active Contour (SOM-RAC) Model [105].* Finally, likewise the SOMCV model, also the SOM-RAC model relies on the global information coming from selected prototypes associated with a SOM, which is trained off-line in an unsupervised way to model the intensity distribution of an image, and used on-line to segment an identical or similar image. In order to improve the robustness of the model, global and local information are combined in the on-line phase, differently from the three models above. The main motivation for the SOM-RAC model is to deal with the sensitivity of local ACMs to the contour initialization (which arise, e.g., when intensity inhomogeneity and additive noise occur in the images) through the combination of global and local information by a SOM-based approach. Indeed, global information plays an important role to improve the robustness of ACMs against the contour initialization and the additive noise but, if used alone, it is usually not sufficient to handle images containing intensity inhomogeneity. On the other hand, local information allows one to deal effectively with the intensity inhomogeneity but, if used alone, it produces usually ACMs very sensitive to the contour initialization. The SOM-RAC model combines both kinds of information relying on global regional descriptors (i.e., suitably selected weights of a trained SOM) on the basis of local regional descriptors (i.e., the local weighted mean intensities). In this way, the SOM-RAC model is able to integrate the advantages of global and local ACMs by means of a SOM.

## 5. Conclusions and Future Research Directions

**Figure 6:**
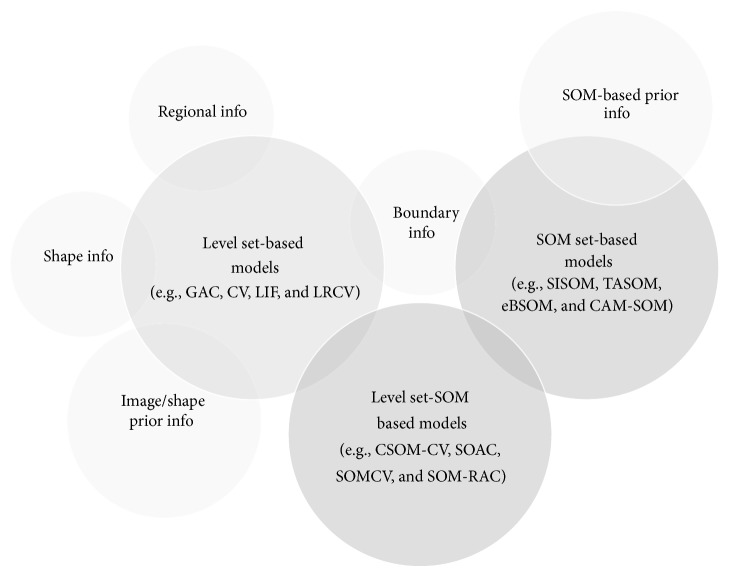
Some relationships between variational level set-based ACMs and Self-Organizing Map (SOM-) based ACMs.

In this paper, a survey has been provided about the current state of the art of Active Contour Models (ACMs), with an emphasis on variational level set-based ACM, Self-Organizing Map (SOM-) based ACMs, and their relationships (see Figure 6).

Variational level set-based ACMs have been proposed in the literature with the aim of handling implicitly topological changes of the objects to be segmented. However, such methods are usually trapped into local minima of the energy functional to be minimized. Then, SOM-based ACMs have been proposed with the aim of exploiting the specific ability of SOMs to learn the edge-map information via their topology preservation property, and reducing the occurrence of local minima of the functional to be minimized, which is also typical of parametric ACMs such as Snakes. This is partly due to the fact that such SOM-based ACMs do not rely on an explicit gradient energy term. Although SOM-based ACMs belonging to the class of edge-based ACMs can effectively outperform other ACM models in handling complex images, most of such SOM-based ACMs are still sensitive to the contour initialization compared to variational level set-based ACMs, especially when handling complex images with ill-defined edges. Moreover, such SOM-based ACMs have not usually the ability to handle topological changes of the objects. For this reason, we have concluded the paper presenting a recently proposed class of SOM-based ACMs, which takes advantage of both SOMs and variational level set methods, with the aims of preserving topologically the intensity distribution of the foreground and background in a supervised/unsupervised way and, at the same time, of allowing topological changes of the objects to be handled implicitly.

Among future research directions, we mention: (1) the possibility of combining, inside SOM-based ACMs, other advantages of variational level set methods in handling the topological changes, in order to obtain a new class of models which are able to handle the topological changes implicitly and, at the same time, to avoid trapping into local minima; (2) the development of more sophisticated supervised/semi-supervised SOM-based ACMs based, for example, on the use of Concurrent Self-Organizing Maps (CSOMs) [91], relying on regional-based information (e.g., local/global statistical information about the intensity, texture, and color distribution) to guide the evolution of the active contour in a more robust way; (3) the possibility of extending current SOM-based ACMs in such a way that the underlying neurons are incrementally added/removed in an automatic way, and suitably trained with the aim of overcoming the limitation of manually adapting the topology of the network, and of reducing the sensitivity of the model to the choice of the parameters; (4) the inclusion of other kinds of prior information (e.g., shape information) in the models reviewed in the paper, with the aim of handling complex images presenting challenging problems such as occlusion; and (5) possible further developments of the machine-learning components of the reviewed models from a streaming-learning perspective, which could lead to a better understanding of video contents through real-time segmentations. Such developments could be obtained by integrating streaming-learning algorithms into the segmentation framework of SOM-based ACMs.

## Figures and Tables

**Table 1:** A summary of the Active Contour Models (ACMs) reviewed in the paper.

ACM	Reference	Regional information		Main strengths/advantages	Main limitations/disadvantages
		Local	Global		
GAC	[24]	No	No	Makes use of boundary information.	Hardly converges in the presence of ill-defined boundaries.
				Identifies accurately well-defined boundaries.	Very sensitive to the contour initialization.

CV	[26]	No	Yes	Can handle objects with blurred boundaries in a global way.	Makes strong statistical assumptions.
				Can handle noisy objects.	Only suitable for Gaussian intensity distributions of the subsets.

SBGFRLS	[68]	No	Yes	Very efficient computationally, and robust to the contour initialization.	Makes strong statistical assumptions.
				Gives efficient and effective solutions compared to CV and GAC.	It is hard to adjust its parameters.

LBF	[71]	Yes	No	Can handle complex distributions with inhomogeneities.	Computationally expensive.
				Can handle foreground/background intensity overlap.	Very sensitive to the contour initialization.

LIF	[32]	Yes	No	Behaves likewise LBF, but is computationally more efficient.	Very sensitive to noise and contour initialization.

LRCV	[31]	Yes	No	Computationally very efficient compared to LBF and LIF.	Very sensitive to noise and contour initialization.

LSACM	[73]	Yes	No	Robust to the initial contour.	Computationally expensive.
				Can handle complex distributions with inhomogeneities.	Relies on a probabilistic model.

GMM-AC	[75]	No	Yes	Exploits prior knowledge.	Makes strong statistical assumptions.
				Very efficient and effective.	Requires a huge amount of supervised information.

SISOM	[38]	No	No	Localizes the salient contours using a SOM.	Topological changes cannot be handled.
				No statistical assumptions are required.	Computationally expensive and sensitive to parameters.

TASOM	[39]	No	No	Adjusts automatically the number of SOM neurons.	No topological changes can be handled.
				Less sensitive to the model parameters compared to SISOM.	Sensitive to noise and blurred boundaries.

BSOM	[93]	No	Yes	Exploits regional information.	Topological changes cannot be handled.
				Deals better with ill-defined boundaries compared to SISOM and TASOM.	Computationally expensive and produces discontinuities.

eBSOM	[94]	No	Yes	Produces smooth contours.	Topological changes cannot be handled.
				Controls the smoothness of the detected contour better than BSOM.	Computationally expensive.

FTA-SOM	[96]	No	Yes	Converges quickly.	Topological changes cannot be handled.
				Is more efficient than SISOM, TASOM, and eBSOM.	Sensitive to noise.

CFBL-SOM	[97]	No	Yes	Exploits prior knowledge.	Topological changes cannot be handled.
				Deals well with supervised information.	Sensitive to the contour initialization.

CAM-SOM	[98]	No	Yes	Can handle objects with concavities, small computational cost.	Topological changes cannot be handled.
				More efficient than FTA-SOM.	High computational cost compared to level set-based ACMs.

CSOM-CV	[102]	No	Yes	Very robust to the noise.	Supervised information is required.
				Requires a small amount of supervised information.	Suitable only for handling images in a global way.

SOAC	[103]	Yes	No	Can handle complex images in a local and supervised way.	Supervised information is required.
				Can handle inhomogeneities and foreground/background intensity overlap.	Sensitive to the contour initialization.

SOMCV	[104]	No	Yes	Reduces the intervention of the user.	Is easily trapped into local minima.
				Can handle multimodal intensity distributions.	Deals with images in a global way.

SOM-RAC	[105]	Yes	Yes	Robust to noise, scene changes, and inhomogeneities.	Very expensive computationally.
				Robust to the contour initialization.	
